# Characterization of a novel PTEN mutation in MDA-MB-453 breast carcinoma cell line

**DOI:** 10.1186/1471-2407-11-490

**Published:** 2011-11-21

**Authors:** Gobind Singh, Leticia Odriozola, Hong Guan, Colin R Kennedy, Andrew M Chan

**Affiliations:** 1 Department of Oncological Sciences, The Mount Sinai School of Medicine, One, Gustave L. Levy Place, New York NY 10029, USA; 2 Department of Pediatrics, Division of Hematology/Oncology/Bone Marrow Transplant, Medical College of Wisconsin, 8701, Watertown Plank Road, Milwaukee WI 53226, USA; 3 Center for Applied Medical Research, Department of Gene Therapy & Hepatology, Avda. Pío XII, 55, 31008-Pamplona, Spain

## Abstract

**Background:**

Cowden Syndrome (CS) patients with germ line point mutations in the *PTEN *gene are at high risk for developing breast cancer. It is believed that cells harboring these mutant *PTEN *alleles are predisposed to malignant conversion. This article will characterize the biochemical and biological properties of a mutant PTEN protein found in a commonly used metastatic breast cancer cell line.

**Methods:**

The expression of PTEN in human breast carcinoma cell lines was evaluated by Western blotting analysis. Cell line MDA-MB-453 was selected for further analysis. Mutation analysis of the *PTEN *gene was carried out using DNA isolated from MDA-MB-453. Site-directed mutagenesis was used to generate a PTEN E307K mutant cDNA and ectopic expressed in PC3, U87MG, MCF7 and *Pten*^-/- ^mouse embryo fibroblasts (MEFS). Histidine (His)-tagged PTEN fusion protein was generated in *Sf9 *baculovirus expression system. Lipid phosphatase and ubiquitination assays were carried out to characterize the biochemical properties of PTEN E307K mutant. The intracellular localization of PTEN E307K was determined by subcellular fractionation experiments. The ability of PTEN E307K to alter cell growth, migration and apoptosis was analyzed in multiple PTEN-null cell lines.

**Results:**

We found a mutation in the *PTEN *gene at codon 307 in MDA-MB-453 cell line. The glutamate (E) to lysine (K) substitution rendered the mutant protein to migrate with a faster mobility on SDS-PAGE gels. Biochemically, the PTEN E307K mutant displayed similar lipid phosphatase and growth suppressing activities when compared to wild-type (WT) protein. However, the PTEN E307K mutant was present at higher levels in the membrane fraction and suppressed Akt activation to a greater extent than the WT protein. Additionally, the PTEN E307K mutant was polyubiquitinated to a greater extent by NEDD4-1 and displayed reduced nuclear localization. Finally, the PTEN E307K mutant failed to confer chemosensitivity to cisplatinum when re-expressed in *Pten*^-/- ^MEFS.

**Conclusions:**

Mutation at codon 307 in PTEN C2 loop alters its subcellular distribution with greater membrane localization while being excluded from the cell nucleus. This mutation may predispose breast epithelial cells to malignant transformation. Also, tumor cells harboring this mutation may be less susceptible to the cytotoxic effects of chemotherapeutics.

## Background

Germ line mutations in the *PTEN *gene are present in 80% of Cowden Syndrome (CS), and in 60% of Bannayan-Riley-Ruvalcaba syndrome (BBRS) [1]. CS patients are predisposed to breast, thyroid, skin, and endometrial malignancies [2]. *PTEN *mutations are found scattered along exon 1 to 8 in both CS and BBRS patients. Significant numbers of mutations (>30%) are found in exon 5 affecting codon 123-131 within the core catalytic domain [3]. However, germ-line mutation in the *PTEN *gene is uncommon in early onset (<40 year old) breast cancer patients with wild-type *BRCA1 *allele [4]. Although the loss of heterozygosity at chromosome 10q23 region has been reported in 17 out of 42 (40%) of invasive breast carcinomas, *PTEN *mutations in sporadic breast carcinomas are rare [5-7]. However, PTEN may still play a role in sporadic breast cancer due to its reduced expression [8].

The *PTEN *gene encodes a 54 kDa lipid phosphatase with specificity towards phosphatidylinositol (3,4,5) triphosphate (PIP_3_) [9]. The 185-amino acid (aa) catalytic domain in the amino(N)-terminus is followed by a phospholipid-binding C2 domain between aa186-353. PTEN C2 domain resembles that of the Ca^2+^-independent membrane recruitment motif found in Protein Kinase C isotypes [10]. While the membrane-facing side of C2 domain is characterized by the polybasic CBRIII loop between aa260-269, the cytosolic-facing portion features an unstructured region of 33-aa between codon 282-314, referred to as the C2 loop [11]. Interestingly, codon Lys289 (K289) within the C2 loop is a target site for mono-ubiquitination and is implicated in nuclear import [12]. Additional ubiquitination sites have also been identified in the N-terminus but these sites are modified by polyubiquitination and play a role in the stability of PTEN through proteosome-mediated degradation [13]. These ubiquitination events have been shown to be catalyzed by NEDD4-1, a ring domain containing E3 ligase [13].

A mutation in the PTEN C2 loop has been identified in a case of CS at codon K289 [12]. The K289E mutation disrupts monoubiquitination and impedes PTEN nuclear import. The resulting loss of growth suppression may explain the numerous intestinal polyps found in this patient. In this manuscript, we report the characterization of another C2 loop mutant uncovered during a screen for aberrant PTEN protein expression in a panel of human breast cancer cell lines.

## Methods

### Cell cultures

All cell lines were obtained from the cell bank of Dr. Stuart Aaronson (Mount Sinai School of Medicine) and maintained in DMEM supplemented with 10% FBS. *Pten*^-/- ^MEFS were a gift from Dr. Hong Wu (UCLA). For transfection of 293T cells, 1 × 10^6 ^cells were transfected in 100-mm plates with 10 μg of DNA using 12 μl of Lipofectamine2000 (Invitrogen) in 6 ml of serum- and antibiotic-free DMEM for 4 h. Cells were lysed after 48 h of incubation. For gene transfer in PC3 cells, 1 × 10^5 ^cells per well in a 6-well plate were transfected with 2-4 μg of DNA using 2 μl of Lipofectamine2000 (Invitrogen) in 1 ml of serum- and antibiotic-free DMEM for 4 h. Cells were analyzed after 48 h incubation. For retroviral gene transfer, 10 μg of pBabe-puro expression vectors was co-transfected with either 10 μg of pCL-ECO or pCL-AMPHO helper viral DNA for *Pten*^-/- ^MEFS and U87MG, respectively. Cultures were infected with predetermined volume of viral supernatants to generate similar ectopic gene expression. After 6 h of incubation, cells were selected in 1.0-1.5 μg/ml of puromycin and marker-selected mass cultures were used in subsequent analysis. For the electroporation of MCF7 cells, approximately 1.5 × 10^6 ^cells were electroporated with 2 μg of expression plasmids using the nucleofection kit according to the manufacturer's instructions (Amaxa). For lipofection of MCF7, approximately 3 × 10^5 ^cells in 6-well plates were transfected with 2 μg of plasmid DNA and 5 μl of lipofectamine 2000 (Invitrogen) for 6 h. Cells were analyzed after 24 to 48 h.

### Sequencing analysis

cDNAs were generated from total RNA of MDA-MB-453 using SuperScript^® ^III First-Strand Synthesis System (Invitrogen). PCR fragments were generated with the following primer pairs covering the entire coding region of *PTEN*: Exon1 and 2: P1 - 5' atgacagccatcatcaaagagatc 3', P8 - 5' aatattgttcctgtatacgccttc 3' Exon3 and 4: P16 - 5' agacttgaaggcgtatacagg 3', P76 - 5' gtcatcttcacttagccattggt 3' Exon5: P26 - 5' cccttttgtgaagatcttgac 3', P41 -5' cagtgccactggtctataatccag 3' Exon6 and 7: P3 - 5' ctggattatagaccagtggcactg 3', P70 - 5' ctgtttgtggaagaactctac 3' Exon8: P71 - 5' gtagagttcttccacaaacag 3', P24 - 5' cagcttcaccttaaaatttgg 3' Exon9: P34 - 5' gccaaccgatacttttctccaaat 3', P2a - 5' tcagacttttgtaatttgtgtatg 3' Amplified fragments were resolved on agarose gels and PCR products were excised and purified by a gel extraction kit (Qiagen). Amplified products were subjected to sequencing analysis using the primers listed above.

### Expression plasmids

All expression plasmids have been reported previously [14-16]. The *PTEN *E307K mutant plasmid was constructed using PCR-based site-directed mutagenesis and cDNA was subcloned in the *Bam*HI and *Eco*RI sites of both pBabe-puro and pCEFL-KZ-AU5 expression vectors.

### Reagents

LY294002 and rapamycin were purchased from Sigma. Cisplatinum was obtained from Sicor, Inc.

### Antibodies

The anti-PTEN mouse monoclonal antibody (A2B1, Santa Cruz), anti-PTEN rabbit polyclonal antibody (#9552, Cell Signaling), anti-AU5 monoclonal antibody (Covance), anti-Akt, anti-p-S473-Akt, anti-p38 antibodies (Cell Signaling), anti-EGFR, anti-SP1, anti-tubulin, and anti-HRP-actin (Santa Cruz) antibodies were purchased from the indicated commercial sources.

### Saponin subcellular fractionation

Cells were solubilized in 10 mM Tris-HCl, pH 7.4, 150 mM NaCl, 0.01% Saponin, 5 mM EDTA, 2 mM EGTA, 1 mM PMSF, 10 μg/ml aprotinin, 10 μg/ml leupeptin, 1 mM Na_3_VO_4_, 1 mM Na_4_P_2_O_7_, and 10 mM NaF for 20 min on ice. The lysates were centrifuged for 30 min at 18,000 × *g*. The supernatant was collected as the cytosolic fraction. The pellet was resuspended in 50 mM HEPES, pH 7.4, 150 mM NaCl, 1% Triton X-100, 1.5 mM MgCl_2_, 1 mM EGTA, 10% glycerol, 1 mM PMSF, 10 μg/ml aprotinin, 10 μg/ml leupeptin, 1 mM Na_3_VO_4_, 1 mM Na_4_P_2_O_7_, 10 mM NaF, and centrifuged for 30 min at 18,000 × *g*. The supernatant was collected as the membrane fraction.

### Nuclear-cytosol fractionation

Nuclear and cytoplasmic fractions were prepared using the NE-PER fractionation kit (Pierce) according to a modified manufacturer's protocol.

### Purification of recombinant PTEN from insect cells

*Sf9 *cells were infected with baculoviruses harboring His-tagged PTEN WT and E307K expression plasmids for 5-7 days. Monomeric PTEN was purified as described previously [16].

### Phosphatase activity assay

Recombinant PTEN was incubated with 50 μM synthetic diC_8_-PI(3,4,5)P_3 _(Echelon) with or without 50 μM synthetic diC_8_-PI(4,5)P_2 _(Echelon), both in soluble form for 10 min at 37°C in a final volume of 25 μl. The reaction buffer was in 10 mM DTT and 25 mM Tris-HCl, pH 8.0. The reaction was stopped with 100 μl of malachite green reagent (Echelon). The amount of phosphate released was measured by reading the absorbance at 620 nm. For measuring PTEN activity *in vitro*, 5 × 10^6 ^293T cells were transfected with 15 μg of expression plasmids using 9 μl of lipofectamine 2000 (Invitrogen). After 48 h, cells were solubilized in 600 μl of a lysis buffer containing 25 mM Tris-HCl, pH 8.0, 150 mM NaCl, 1% Triton X-100, 2 mM DTT, 1 mM EDTA, 1 mM PMSF, 10 μg/ml aprotinin, and 10 μg/ml leupeptin. Approximately 3 mg of total cell extracts were immunoprecipitated with 5 μg of an anti-AU5 antibody for 4 h and immunocomplexes were affinity absorbed onto 30 μl of GammaBind G sepharose beads. PTEN phosphatase activity was then assayed as described above.

## Results

### Identification of PTEN E307K mutation in MDA-MB-453

**Figure 1:**
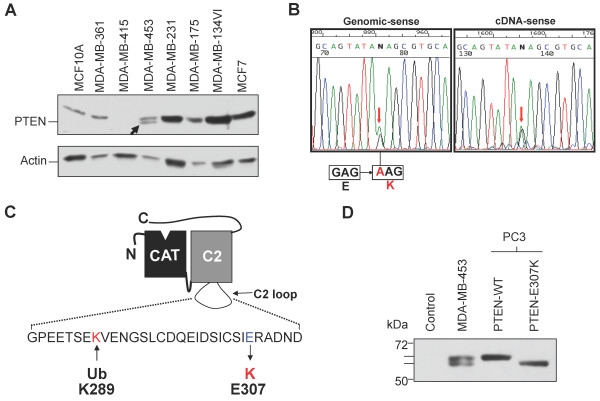
**Aberrant PTEN protein species in MDA-MB-453**. **(A) **Western blotting analysis of PTEN in human breast cancer cell lines. The faster mobility species in MDA-MB-453 is highlighted with an *arrow*. Actin was used as a loading control. **(B) **Sequencing analysis of exon8 of PTEN using genomic DNA and cDNA derived from MDA-MB-453 showing the G to A substitution at codon 307 altering the glutamate (*E*) residue to a lysine (*K*). **(C) **Schematic representation of PTEN depicting the catalytic domain (CAT) and the C2 loop with the ubiquitination (Ub) site at K289 and the E307K mutation shown. **(D) **Both wild-type (*WT*) and E307K mutant were transfected in a PTEN-null cell line, PC3. Note the faster migrating E307K mutant. All membranes were probed with the monoclonal anti-PTEN antibody (A2B1).

During the examination of a panel of human breast cancer cell lines for aberrant PTEN expression, we detected an additional band that migrated at a smaller apparent molecular weight (~52 kDa) than the WT PTEN protein (~54 kDa) in the MDA-MB-453 cell line (Figure 1 A). Short Tandem Repeat (STR) analysis confirmed the authenticity of this cell line as MDA-MB-453 (data not shown). Sequencing analysis of the entire *PTEN *coding region has identified a single G to A mutation at codon 307 altering the negatively-charged glutamate (GAA) to a positively-charged lysine (AAA) residue (referred to as E307K hereafter) (Figure 1 B). The same mutation was also detected in both genomic and complementary DNA (cDNA) of this cell line (Figure 1 B). Amino acid E307 was located within the C2 loop of PTEN and was in proximity to the monoubiquitination site at K289 (Figure 1 C). Two separate groups have previously identified the E307K mutation in MDA-MB-453 cell line [17,18], and not in other breast cancer cell lines examined in this study [19]. However, both groups have not further characterized the biological properties of this PTEN E307K mutant.

To confirm that the faster migrating PTEN band in MDA-MB-453 represented the PTEN E307K mutant, we engineered a *PTEN *E307K mutant cDNA and ectopically expressed in a PTEN-null cell line, PC3. As expected, the PTEN E307K mutant displayed a similar mobility as the faster migrating species found in MDA-MB-453 (Figure 1 D). By both mass spectroscopy and phosphatase treatment, we failed to identify evidence of post-translational modifications of the PTEN E307K mutant protein (data not shown). Consistently, E307K gene products synthesized by *in vitro *translation reactions, which presumably devoid of all modification events, still retained a faster motility (data not shown). Therefore, we conclude that the glutamate to lysine substitution at codon 307 resulted in a change in the charge of PTEN and this caused an aberrant mobility on SDS-PAGE gel. This mobility shift greatly facilitated the ability to differentiate between WT and E307K proteins in subsequent studies.

### Biochemical and growth suppressing activities of PTEN E307K

**Figure 2:**
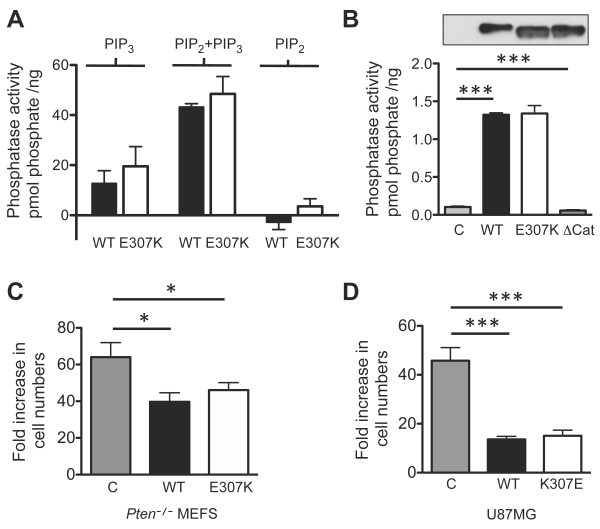
**Biochemical and biological properties of PTEN E307K mutant**. **(A) **The phosphatase activity of the indicated PTEN isoforms was measured in the presence or absence of PIP_2 _using the malachite green phosphatase assay with PIP_3 _as the substrate. **(B) **Vector control (*C*), WT, E307K, and ΔCat expression plasmids were transfected in 293T cells. PTEN proteins were immunoprecipitated with an anti-AU5 antibody and immobilized on sepharose beads. Phosphatase activity was measured in triplicates using the malachite green assay with PIP_3 _as the substrate. The relative levels of PTEN isoforms in the immunoprecipitates were monitored by a polyclonal anti-PTEN antibody (*insert*). Similar data was reproduced in an additional experiment. **(C) **Vector control (*C*), WT, and E307K cDNAs were transduced into *Pten*^-/- ^MEFS (*left panel*) and U87MG (*right panel*) cells through retroviral-mediated gene transfer. Puromycin (1 μg/ml) resistant mass cultures were analyzed for relative proliferative capacity after 6 days in cultures. The fold-increase in cell numbers was measured by cell counting. Data represents triplicated measurements from a single experiment. *Bars*, SD. *, p < 0.05. ***, p < 0.0005.

To investigate if the PTEN E307K mutant displayed altered enzymatic activity, WT and E307K proteins were epitope-tagged with 6xhistidine and expressed in *Sf9 *insect cells. Affinity purified recombinant proteins were subjected to lipid phosphatase assay in the presence of combinations of PIP_2 _and PIP_3_. As shown in Figure 2 A, while PTEN proteins did not catalyze the release of phosphate when PIP_2 _was used as a substrate, both WT and E307K catalyzed either PIP_3 _or PIP_2 _+ PIP_3 _to a similar extent. To validate this result, both WT and E307K PTEN cDNAs were tagged with an AU5 epitope and transiently transfected in 293T cells. As a negative control, a D92A/C124A catalytic-dead (ΔCat) mutant was also included. All PTEN isoforms were immunoaffinity purified with an anti-AU5 monoclonal antibody and subjected to phosphatase assay using PIP_3 _as a substrate. Similar to recombination proteins, both WT and E307K displayed very similar lipid phosphatase activity while the ΔCat mutant failed to elicit any enzymatic activity (Figure 2 B).

To address if the lack of differences in enzymatic activity between WT and E307K proteins could be extended to their tumor suppressing activities, both cDNAs were expressed in both *Pten*^-/- ^MEFS and a PTEN-null glioblastoma cell line, U87MG, through retroviral-mediated gene transduction. As shown in Figure 2 C, both WT and E307K were able to exert ~40% and ~75% growth inhibitory effects on *Pten*^-/- ^MEFS and U87MG, respectively. From these data, we conclude that PTEN E307K mutant does not display observable defects in both intrinsic lipid phosphatase activity and growth suppressing potential in PTEN-null cells.

### PTEN E307K mutant displays higher membrane localization

**Figure 3:**
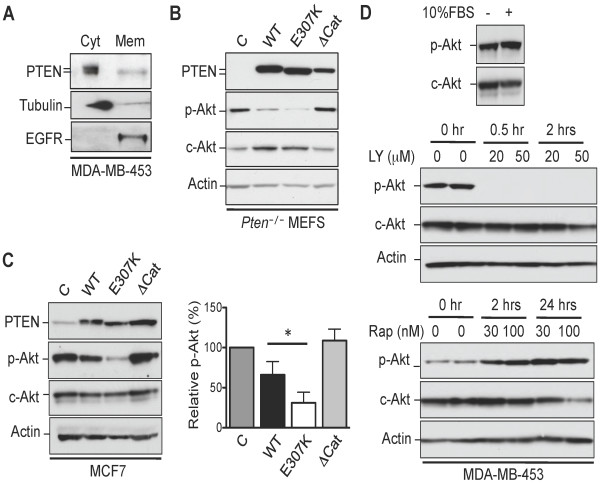
**Suppression of Akt by PTEN E307K mutant**. **(A) **MDA-MB-453 cells were solubilized in 0.01% saponin-containing buffer. The membrane (*Mem*) and cytosolic (*Cyt*) fractions were resolved on SDS-PAGE gel and probed with an anti-PTEN monoclonal antibody (A2B1). The purity of the cytosolic and membrane fractions was confirmed with antibodies against tubulin and EGFR, respectively. **(B) ***Pten*^-/- ^MEFS were transduced with the indicated retroviral expression plasmids. Cell lysates were prepared from cell lines and subjected to Western blotting analysis using the indicated antibodies. **(C) **MCF7 cells were electroporated with the indicated expression plasmids and total cell extracts were prepared for Western blotting analysis. Results from three independent experiments were quantified (*right panel*). *Bars*, SE. *, p < 0.05. **(D) **MDA-MB-453 cells were cultured in the presence or absence of 10% FBS. Cell extracted were prepared and the levels of p-Akt and c-Akt were monitored by Western blotting analysis (*upper panel*). MDA-MB-453 cells were treated with LY294002 (*middle panel*) or rapamycin (*lower panel*) at the indicated concentrations and durations. The activation states of Akt were monitored by Western blotting analysis with actin as a loading control.

Since the C2 domain of PTEN has been implicated in membrane targeting [10], attempts were made to examine the level of PTEN E307K on the cell membrane relative to WT protein. Taking advantage of the mobility difference between WT and E307K on SDS-PAGE, MDA-MB-453 cells were fractionated into cytosolic and saponin-solubilized membrane fractions. We have previously used this method to investigate the membrane localization of different PTEN mutants [16]. As shown in Figure 3 A, the faster migratory species, which represented the E307K mutant, was readily detectable in the membrane fraction that was co-localized with EGFR. On the contrary, the slower migrating species, which represented the WT protein, was almost undetectable. This data is consistent with the transient nature of PTEN at the plasma membrane reported previously [20]. As a lipid phosphatase, the higher level of PTEN E307K on the cell membrane would predict a greater suppression of the PI3-K signaling. To test this, *Pten*^-/- ^MEFS stably expressing WT and E307K were analyzed for the activation state of Akt. As predicted, while WT PTEN repressed p-Akt level by ~4.5-fold, the E307K mutant displayed a great suppression of ~6-fold (Figure 3 B). Of note, both WT and the E307K proteins were expressed to a similar level. Since MDA-MB-453 was derived from a breast carcinoma, we sought to express the E307K mutant in a breast cancer cell line. For this, WT and E307K expression plasmids were electroporated in a PTEN-positive MCF7 cell line. Similar to *Pten*^-/- ^MEFS, the E307K mutant was significantly more effective in attenuating p-Akt level than WT protein (Figure 3 C). This effect was unlikely to be an artifact of overexpression as the levels of ectopic PTEN were only between 3-5 fold. Also, since electroporation only achieved 60-70% transfection efficiency, the ability of E307K mutant in suppressing p-Akt could have been greater.

Since both WT and the E307K mutant were co-expressed in MDA-MB-453, it would be necessary to evaluate the activation state of the PI3-K signaling pathway in this cell line. Serum deprivation did not result in altered p-Akt suggesting that the PI3-K pathway was constitutively active (Figure 3 D, *upper panel*). Treatment with the PI3-K inhibitor, LY294002, resulted in the complete abolishment of p-Akt signals within 30 mins. In addition, treatment with the mTOR inhibitor, rapamycin, stimulated Akt activation through the inactivation of an S6K-mediated negative feedback loop [21]. Thus, PI3-K pathway is constitutively active in MDA-MB-453 due most likely to an oncogenic event located at or upstream of PI3-K.

### PTEN E307K mutant has enhanced polyubiquitination

**Figure 4:**
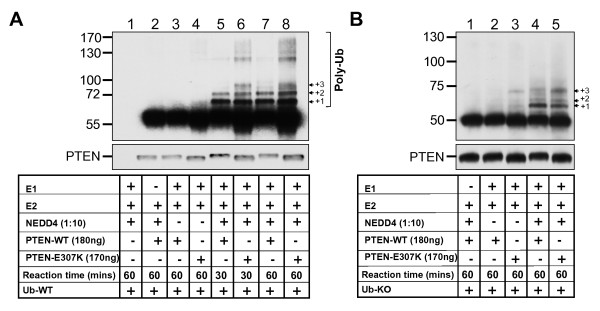
**Ubiquitination of PTEN E307K by NEDD4-1**. Recombinant WT and E307K PTEN were incubated with either Ub-WT **(A) **or Ub-KO **(B) **under the conditions indicated. Reactions were resolved on SDS-PAGE and probed with an anti-PTEN polyclonal antibody (#9552). The amount of PTEN in each reaction is shown (*lower panels*).

The PTEN C2 loop is an unstructured domain harboring a single monoubiquitination site at K289 [13]. With the close proximity to E307, we speculated that the Glu to Lys substitution at codon307 might constitute a *de novo *ubiquitination site and this prompted us to test if PTEN E307K displayed aberrant ubiquitination pattern. For this, we have tested the ability of NEDD4-1, a PTEN E3 ligase [13] in mediating ubiquitination of PTEN recombinant proteins purified from baculovirally-infected *Sf9 *insect cells. As shown in Figure 4 A, NEDD4-1 catalyzed a more extensive polyubiquitination of E307K as early as 30 minutes when compared to WT (Figure 4 A, compare *lane 5 and 6*). Also, an additional polyubiquitinated species at +3 position was detected only in the E307K protein (Figure 4 A, lane 6). Furthermore, when similar reaction was conducted in the presence of a defective ubiquitin (Ub-KO), a E307K (+3) species at ~72 kDa was already observed when only E1 and E2 components were present (Figure 4 B, lane 3). This could be due to residual E3 ligase activity co-purified from *Sf9 *as has been reported before [13]. Since Ub-KO could not be polyubiquitinated, the number of bands signifies the number of ubiquitination site. Based on these results, both WT and E307K have three Ub sites. More significantly, the addition of E3 enhances the intensity of the +3 band in E307K when compared to WT. Thus, we conclude from these studies that E307K promotes ubiquitination. We further hypothesize that the preferential ubiquitination at E307K may set up a "decoy" effect, potentially disrupting monoubiquitination at K289 and nuclear import.

### PTEN E307K mutant displays reduced nuclear localization

**Figure 5:**
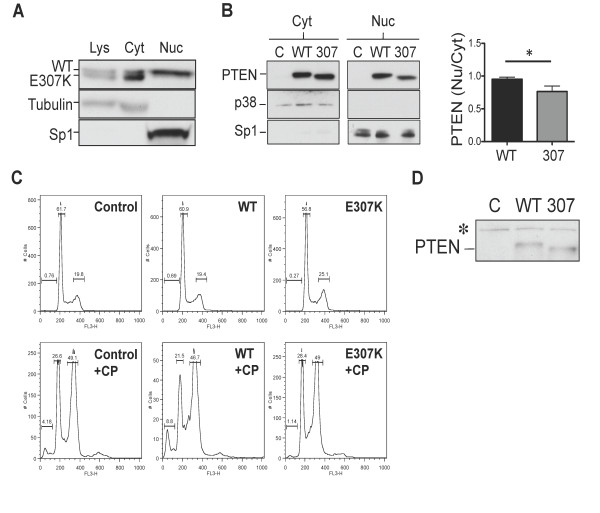
**Aberrant nuclear localization of PTEN E307K mutant**. **(A) **Total cell lysate (*Lys*) from MDA-MB-453 was fractionated into cytosolic (*Cyt*) and nuclear (*Nuc*) fractions with the NE-PER kit (Pierce, IL). Tubulin and Sp1 were used as cytosolic and nuclear markers, respectively. Similar results were obtained from two independent experiments. **(B) **MCF7 cells were transfected by lipofection with the indicated expression plasmids and cell lysates were fractionated after 24 h. Purity of the cytosolic and nuclear fractions was confirmed with p38 and Sp1, respectively. Results represent data from two experiments performed in duplicates. *Bars*, SE. *, p < 0.05, one-tailed t-test (*right panel*). **(C) ***Pten*^-/- ^MEFs expressing WT and E307K were treated with PBS (*upper panels*) or 5 μM cisplatinum (*+CP*)(Sicor Inc., CA) for 48 hours. Cells were stained with PI. Note the reduction in the % of sub-G1 cells in E307K+CP. **(D) **Western blotting analysis of PTEN expression. Anti-PTEN polyclonal antibody (#9552) was used in all panels. Abbreviations: *C*, control. *, background signals.

A previous study has identified K289 as a site for monoubiquitination and important for PTEN nuclear targeting capacity [13]. The close proximity of E307K to K289 and the aberrant ubiquitination pattern observed with E307K have prompted us to investigate if the E307K mutant altered nuclear localization. MDA-MB-453 cell extract was fractionated into cytosolic and nuclear fractions. As shown in Figure 5 A, the slower migrating species, which represented the WT protein, was the only prominent band in the nuclear fraction. On the contrary, both WT and E307K mutant were present in the cytosolic fraction with the E307K protein at relative higher level than WT. This data suggests that the E307K mutant is defective in nuclear import. To validate this result, PTEN cDNAs were transiently transfected in MCF7 breast cancer cells and subjected to subcellular fractionation. The nuclear-to-cytosol ratio was reduced by 19.7% for the E307K mutant when compared to the WT protein (Figure 5 B). Statistical analysis revealed a significant difference only when one-tailed t-test was used to predict a decrease in nuclear localization. However, no significant difference was obtained with two-tailed analysis. This marginal difference could be due to cell-type specific differences between MDA-MB-453 and MCF7.

Previous studies have alluded to the ability of nuclear PTEN in regulating genome integrity and growth suppression [22-25]. The fact that the E307K mutant was excluded from the nuclear compartment has led us to examine if cells expressing this PTEN mutant could have altered sensitivities to conventional chemotherapeutic agent. To test this, *Pten*^-/- ^MEFS stably expressing WT and the E307K mutant were tested. Under untreated conditions, all these cells have undetectable sub-G1 cells, which were presumably the apoptotic fraction (Figure 5 C). Exposure to 5 μM cisplatinum resulted in the appearance of 4% of sub-G1 apoptotic cells in parental *Pten*^-/- ^MEFS. On the other hand, the re-expression of WT PTEN increased this to 8.8%. However, the fraction of apoptotic cells was only 1.1% in E307K expressing cells. This difference was unlikely to be the result of protein expression since both WT and the E307K mutant were expressed to a similar level (Figure 5 D). Thus, these results implicate a role for E307K in conferring chemoresistance.

## Discussion

In this report, we have characterized a unique PTEN C2 loop mutant present in a widely used human metastatic carcinoma cell line, MDA-MB-453. This tumor was originated from a 42 year old female diagnosed with breast cancer in 1970 at MD Anderson Cancer Center [26]. A right mastectomy was performed followed by radiation and chemotherapy. Tumor relapse was recorded in 1974 and after further treatments, the patient died from the disease in 1976. MDA-MB-453 represents a molecular apocrine breast cancer, which is estrogen receptor-negative but androgen receptor-positive, and is commonly detected in patients with CS [27,28]. However, we do not have evidence that the E307K mutation represents a germ line mutation similar to those found in CS patients. The lack of access to germ line tissues hampers our efforts in addressing this possibility.

Mutations in PTEN C2 loop are not without precedent. As mentioned above, a K298E mutation was uncovered in a CS patient and the nuclear PTEN levels in the dysplastic intestinal polyps were reduced [12]. More interestingly, PTEN C2 loop is highly acidic in nature with 10 out of 32 (~31%) amino acids are either glutamate or aspartate residues. The substitution with a basic lysine residue at E307 is expected to introduce a considerable change to the charge surfaces. Indeed, a search of the Sanger Tumor Gene Mutation Database has identified similar E to K substitutions at codons E284, E288, and E291. Coincidentally, these mutations were either from endometrial or vulva cancers [29-31]. This cluster of E to K mutations highlights the likelihood that C2 loop mutations play a role in either the initiation or progression of certain human malignancies. It is also note worthy that there was no evidence of a loss of heterozygosity in the MDA-MB-453 tumor as the WT protein species is expressed to a similar level. Whether the E307K mutant can act as a dominant acting oncogene or has dominant inhibitory effects on the WT counterpart is currently unknown. Alternatively, haploinsuffiency may play a role in the progression of the MDA-MB-453 tumor as has been demonstrated for the *PTEN *tumor suppressor gene [32].

The E307K mutant displays similar lipid phosphatase activity as the WT protein. This is not surprising since eliminating the 24-aa C2 loop between aa286-309 did not significantly altered PTEN enzymatic activity and membrane binding affinity [11]. The finding of a higher level of the E307K mutant in the saponin soluble membrane fraction is intriguing. One possibility is that the substituted lysine may serve as a *de novo *binding interface with the negatively charged membrane. Previously, our group and others have identified phosphorylation sites in the PTEN C-terminal tail that may serve to mask C2 domain from the plasma membrane [16,33,34]. One speculation is that E307K may serve as a decoy binding site for C-terminal tail and thereby unmasking the C2 domain for membrane targeting. Demonstrating this would need biophysical evidence but will pose considerable challenge due to the unstructured nature of the C2 loop [11].

The greater membrane localization of the E307K mutant is consistent with the observation of greater suppression of p-Akt levels in both *Pten*^-/- ^MEFS and MCF7 cells. This finding is, however, inconsistent with the fact that E307K shows similar growth suppressing activity as WT. A plausible explanation is that Akt activation does not correlate with cell proliferation in *Pten*^-/- ^MEFS. Alternatively, the threshold of repressed p-Akt level sufficient for growth suppression is rather low for differences to be registered. Also, it is counterintuitive that breast cancer cells would acquire a mutation in PTEN that has greater ability in suppressing the PI3-K pathway. At present, we do not have a solid explanation to this paradox. One speculation is that since Akt1 has been shown to block breast cancer cell migration [35,36], the presence of a E307K mutant may repress Akt1 activation and promote metastatic growth. An alternative explanation is that the persistent suppression of PI3-K > Akt pathway in cells harboring the E307K mutant may promote secondary mutations that can subvert this repression. Indeed, MDA-MD-453 harbors a H1047R oncogenic mutation in the *PIK3CA *gene [18]. This is consistent with the fact that the PI3-K inhibitor, LY294002, could completely abolish p-Akt in this tumor line.

The heightened polyubiquitination observed with the E307K mutant reaffirms the role of C2 loop in the post-translational modification of PTEN. However, it is not clear which sites are being modified. It is tempting to speculate that the aberrant polyubiquitination of E307K can result in PTEN nuclear exclusion since monoubiquitination appears to be critical for nuclear import [12]. In addition, the increase in polyubiquitination does not result in greater degradation of the PTEN E307K protein since its level is almost identical to that of WT.

Several cellular functions have been ascribed to nuclear PTEN including p53-dependent oxidative stress response [22], protection against DNA double strand breaks [23], and growth suppression through binding to the APC-CDH1 complex [37]. Currently, it is unclear if PTEN E307K mutant binds p53 or APC-CDH1 with altered affinity. The inability of PTEN E307K mutant in sensitizing cells to cisplatinum would argue for a negative role of nuclear PTEN in promoting DNA damage response. Given the pro-survival function of Akt, our data is inconsistent with the fact that PTEN K307E mutant possesses a greater ability in suppressing Akt activation in *Pten*^-/- ^MEFS. To resolve these issues, clearly defining the Akt isoforms being regulated would be important. To clearly define the role of E307K mutation, it may be necessary to eliminate this mutant *PTEN *allele by either gene silencing or somatic gene knockout in MDA-MB-453. In summary, this study provides an initial characterization of a PTEN C2 loop mutant present in a breast cancer cell line. To our knowledge, MDA-MB-453 is the only known cell line that harbors a PTEN C2 loop mutation. Thus, this study highlights the availability of a commonly used breast cancer cell line for future research into the role of PTEN C2 loop in nuclear/cytosol partitioning.

## Conclusions

We have identified a mutation within the C2 loop of PTEN in a human metastatic breast cancer cell line, MDA-MB-453. The E307K mutant protein displays subtle changes in biochemical and biological properties unlike other PTEN mutations that inactivate its catalytic activity. The major defect is associated with aberrant subcellular distribution of the cytosolic pool of PTEN, being preferentially targeted to the plasma membrane but excluded from the cell nucleus. These changes may predispose breast epithelial cells to transformation and the acquisition of chemoresistance. This mutation is highly reminiscent to those subtle C2 loop mutations found in CS patients. These findings also highlight the importance of future work in further unraveling the tumor suppressing effects of nuclear and membrane PTEN and how their interplay is differentially regulated in different cancer types. As a widely used metastatic breast carcinoma cell line, MDA-MB-453 will be a valuable resource for future studies related to the regulation of PTEN subcellular distribution.

## Competing interests

The authors declare that they have no competing interests.

## Authors' contributions

All authors read and approved the final manuscript. GS made the observations of the nuclear exclusion and membrane localization of the E307K mutant in MDA-MB-453. LO performed the purification and lipid phosphatase assays. HG, and CRK performed both growth assays and Western blotting analysis. AMC analyzed the data and wrote the manuscript.

## Pre-publication history

The pre-publication history for this paper can be accessed here:

http://www.biomedcentral.com/1471-2407/11/490/prepub
